# Two sisters in the same dress: *Heliconius *cryptic species

**DOI:** 10.1186/1471-2148-8-324

**Published:** 2008-11-28

**Authors:** Nathalia Giraldo, Camilo Salazar, Chris D Jiggins, Eldredge Bermingham, Mauricio Linares

**Affiliations:** 1 Instituto de Genética, Universidad de los Andes, Carrera 1 No 18a – 70, P.O. Box 4976, Bogotá D.C, Colombia; 2 Department of Zoology, University of Cambridge, Downing Street, Cambridge, CB2 3EJ, UK; 3 Smithsonian Tropical Research Institute, Apartado 0843-03092, Panamá, República de Panamá

## Abstract

**Background:**

Sister species divergence and reproductive isolation commonly results from ecological adaptation. In mimetic *Heliconius *butterflies, shifts in colour pattern contribute to pre- and post-mating reproductive isolation and are commonly correlated with speciation. Closely related mimetic species are therefore not expected, as they should lack several important sources of reproductive isolation.

**Results:**

Here we present phenotypic, behavioral and genetic evidence for the coexistence of two sympatric 'cryptic' species near Florencia in the eastern Andes of Colombia that share the same orange rayed colour pattern. These represent *H. melpomene malleti *and a novel taxon in the *H. cydno *group, here designated as novel race of *Heliconius timareta*, *Heliconius timareta florencia*. No-choice mating experiments show that these sympatric forms have strong assortative mating (≈96%) despite great similarity in colour pattern, implying enhanced divergence in pheromonal signals.

**Conclusion:**

We hypothesize that these species might have resulted from recent convergence in colour pattern, perhaps facilitated by hybrid introgression of wing pattern genes.

## Background

Ecological selection is known to play an important role in speciation [1,2]. Where ecological traits under divergent selection also affect the species recognition system, speciation can be rapid [3,4]. Young species pairs provide evidence for this, such as stickleback morphs found in glacial lakes in which body size diverges through adaptation to different niches and is correlated with mate choice [5,6]. Similarly, in Darwin's finches adaptive variation in beak size also promotes reproductive isolation through pleiotropic effects on song [7,8]. In many butterflies colour pattern similarly plays a role in mate recognition [9,10] and is also under ecological selection for signaling to predators, crypsis and thermoregulation [11].

In *Heliconius *butterflies shifts in colour pattern have been shown to play a major role in speciation [12,13]. Closely related species and sub-species typically differ in colour pattern [14] and are adapted to local Müllerian mimicry rings in which distasteful species converge on a common pattern [15,16]. The pattern differences between related forms lead to strong assortative mating [12,13]. Frequency dependent selection contributes to maintaining the mimetic patterns and also causes post-mating isolation [17,18]. Thus, between closely related species such as *H. cydno *and *H. melpomene*, rare hybrid individuals are likely to be strongly selected against, as their pattern will not be recognized by predators [12,15]. These species overlap extensively across Central America and the Andes [19,20]. Through their geographical range, *H. melpomene *mimics *H. erato *with a black background and red, yellow and orange marks, whereas *H. cydno *mimics species of the *H. sapho *group with a black-blue background and white and yellow marks [12,21]. Although *H. melpomene *and *H. cydno *occasionally hybridize in nature (less than 0.1%), reproductive isolation is strong, with ecological isolation and colour pattern associated mate choice playing the major role [12,22,23].

The phylogeny of *Heliconius *supports a key role for pattern change in speciation, with almost all sister species differing in colour pattern [24]. Exceptions such as *H. sara *and *H. leucadia *are far more genetically divergent than species in the *H. melpomene *clade, implying that speciation was relatively ancient [24]. It was therefore a surprise when a putative 'cryptic' species was identified in the *H. cydno/melpomene *species complex, implying either mimetic convergence between closely related species, or speciation without pattern change [25]. *H. tristero *was described as a new species from the southeastern Andes of Colombia with a cydno-like mtDNA haplotype, but a black, red and yellow "postman" pattern similar to sympatric *H. melpomene mocoa *[25]. This species was initially met with scepticism, mainly due to the fact that only two individuals of *H. tristero *were collected, combined with the likelihood of mtDNA introgression between *H. cydno *and *H. melpomene *[26]. It was therefore suggested that the *H. tristero *specimens most likely represent rare hybrids between *H. cydno *and *H. melpomene*.

**Figure 1:**
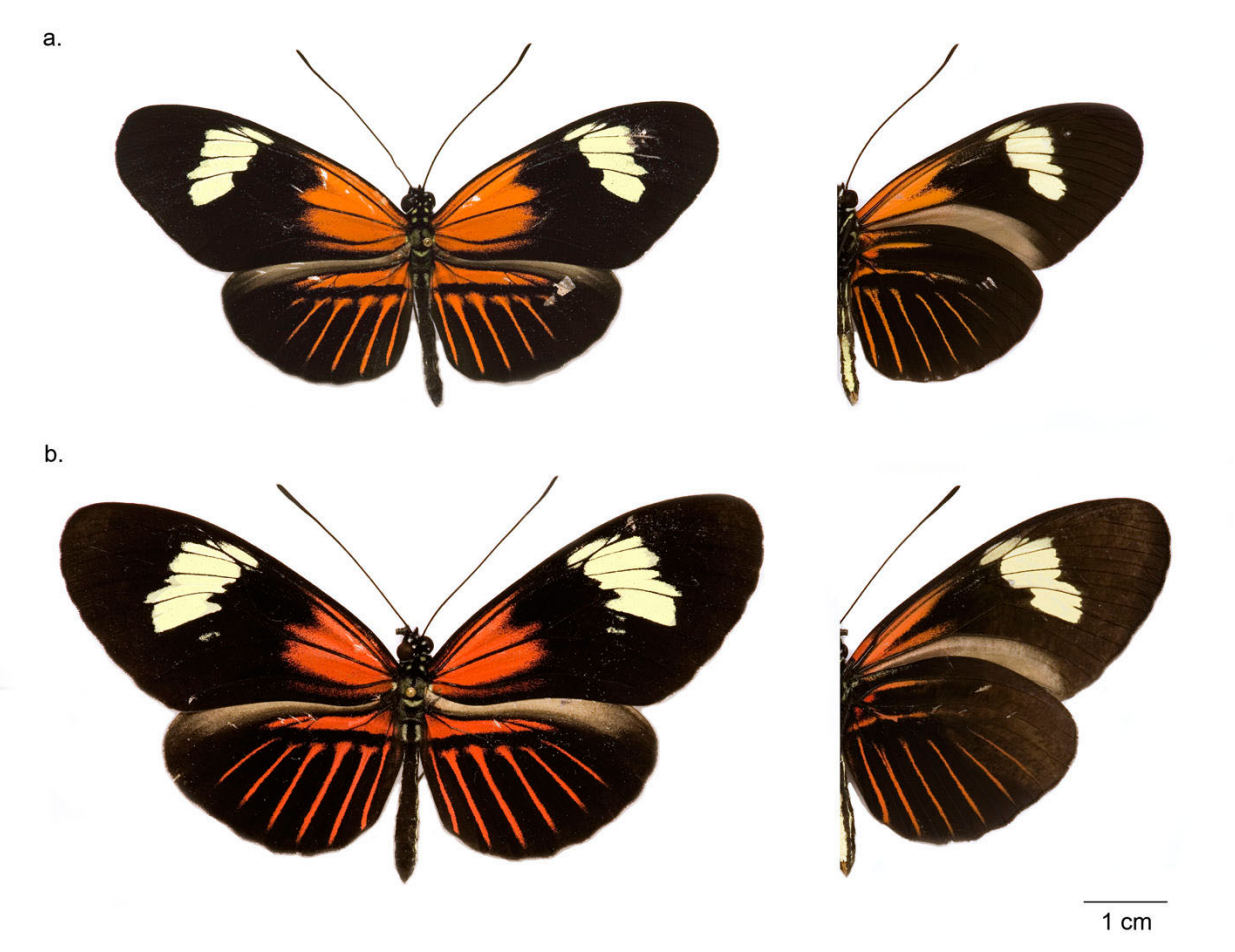
**Species under study**. *a. H. m. malleti *from Florencia and *b*. its co-mimetic *H. timareta florencia *(Holotype). Left side: dorsal view, right side: ventral view. Both individuals are lodged at the Natural History Museum ANDES of Universidad de los Andes in Bogotá, Colombia (accession numbers: Andes-L-01370 and Andes-L-01369).

The discovery of very closely related sympatric mimetic forms is therefore of considerable interest as it would imply either speciation without colour pattern shifts, or alternatively very recent mimetic convergence between hybridizing species, possibly through adaptive introgression [23,26]. Here we present compelling evidence of a cryptic species pair, in which an *H. cydno *cognate resembles and coexists in sympatry with a well-known race of *H. melpomene *(Figure 1).

## Methods

### Sampling and species assignment

A total of 196 adult butterflies were collected between 2002–2006 in Sucre (01°48'12¨N 75°39'19¨W) near Florencia, Colombia. For comparison of larval and adult morphology, an additional 118 butterflies representing *H. cydno weymeri *(N = 21), *H. c. cordula *(N = 19), *H. c. zelinde *(N = 12), *H. melpomene vulcanus *(N = 17), *H. m. melpomene *(N = 26) and *H. m. mocoa *(N = 23) were collected from various sites in Colombia. Some of the individuals from Florencia were maintained alive in separate insectaries at La Vega, Cundinamarca for mate and host choice experiments. Wings were removed from the remaining specimens and bodies preserved in DMSO or Ethanol (96%) for subsequent phenotypic (N = 131) and molecular analysis (N = 142) and stored in the permanent collection of the Instituto de Genética of Universidad de los Andes in Colombia (the collection is now part of the recently created Natural History Museum ANDES). DNA extractions were made from one-third of the thorax of each individual using the DNeasy tissue Kit (QIAGEN), following the manufacturers' protocol. Digital images were obtained by scanning the wings and quantitative measurements taken using *TpsDig *image software [27]. One-way Analyses of Variance (ANOVA) on phenotypic measurements were performed with SPSS 11.0.4 software [28].

### No-choice mating experiments

Once diagnostic characters for the two morphotypes had been established we carried out mate choice experiments to investigate reproductive isolation. No-choice mating trials were performed in 2 m × 2 m × 2 m insectaries in La Vega, Cundinamarca between June 2004 and October 2005. These experiments are a simulation of a natural situation where males encounter females singly, and estimate the reluctance of both sexes to mate inter-specifically. A virgin female (one to three days old) of each morphotype was presented to ten mature males (more than 10 days old) in a single insectary for two days. Successful and failed matings were recorded every 30 minutes between 6 am to 2 pm. Males were used only once. In order to detect matings that were unobserved, females were checked for the presence of a spermatophore in their reproductive tract. A binomial mating probability P_ixj _and 95% confidence intervals were obtained for each combination of *i*-type female and *j*-type male using Maximum Likelihood as previously described [29].

### Host plant choice and larval morphology

Females of both Florencia populations (*H. m. malleti *and *H. cydno *cognate) and other populations of the two species were kept in individual insectaries with known host plants, *Passiflora edulis*, *P. maliformis*, *P. ligularis*, *P. arborea*, *P. quadrangularis*, *P. oerstedii *[30,31], and as controls two species used by *H. erato*, *P. suberosa*, and *P. rubra *[32]. The number of eggs laid per plant by each female was recorded twice a week. Offspring from the same females were used for analysis of larval morphology. *H. melpomene *and *H. cydno *larvae are distinguishable by cephalic colour [14] which is pale yellow in *H. melpomene *and orange in *H. cydno*. Pictures of larvae raised from wild females were taken under similar light conditions with a colour standard. Between four and seven larvae were analysed from each of 21 females collected in Florencia and compared to those from other localities. Pictures were processed using Scion Image (Scion Corporation, Frederick, MD, USA) and four RGB indexes calculated, R' = r/(r+g+b), G' = g/(r+g+b), B' = b/(r+g+b) and LM = R'-G' [33]. Statistical significance of differences in indices was tested with one-way variance analysis using SPSS 11.0.4 software [28].

### Sequence Analysis

We sequenced a region of nuclear DNA spanning the 3' end of the Z-linked *Triose phosphate isomerase *(Tpi) gene, from thirteen female specimens of *H. m. malleti *and seven *H. cydno *cognate from Sucre Florencia, Caquetá and nine specimens of *H. timareta *from Ecuador (additional file 1). The Polymerase Chain Reaction (PCR) was performed using primers and conditions outlined elsewhere [34]. The PCR products were electrophoretically separated on 1.5% low melting point agarose (Invitrogen), and the bands were cut from the gel and dissolved in gelase (Invitrogen). Clean PCR products were sequenced using the DNA sequence Kit (Big Dye 3.1, PE Applied Biosystems), in an ABI Prism 3100 Genetic Analyzer (PE Applied Biosystems).

We also sequenced fragments of two mitochondrial genes (COI-COII) from seventeen *H.m. malleti*, seven *cydno*-like and seven *H. timareta *individuals. In addition to sequences obtained here, we also included *CO *and *Tpi *sequences from GenBank (additional file 1). PAUP* v4.0b10 [35] was used to search for a maximum parsimony tree, using a heuristic search with TBR branch swapping; bootstrap values were calculated with 5000 replicates using the same search conditions.

MrModeltest v2.2 [36] was used to determine the most appropriate model of nucleotide substitution based on hierarchical likelihood ratio tests. For the mtDNA COI/COII data set MrModeltest identified the GTR+I+G model [37,38], and for *Tpi*, the GTR+G model [37,38]. Bayesian phylogenetic analyses were performed with MrBayes v3.1 [39] following the analytical recommendations of the authors [40]. The base frequency parameters determined by MrModeltest were used for analysis in MrBayes v3.1, with remaining parameters estimated using the GTR+I+G model for COI/COII and GTR+G for *Tpi*. Four differentially heated Markov chains were initiated from random trees, run for 10^6 ^generations and sampled every 100 cycles. Likelihood values were plotted against number of generations to determine the points at which stationary was reached. All trees sampled before these points were discarded and the remaining tree samples were used to generate a 50% majority rule consensus tree (*n *= 12101 for COI/COII and *n *= 9981 for *Tpi*). The posterior probability of each clade is provided by the percentage of trees identifying the clade [39,40].

### Microsatellite Analyses

A total of 13 microsatellite loci were amplified and scored as described previously [41]. Hardy-Weinberg equilibrium and linkage disequilibrium and their significance were tested for at each locus using Arlequin v2000 [42]. Two Bayesian model-based clustering algorithms implemented in the programs STRUCTURE 2.2 [43] and BAPS 4 [44] were used to test the hypothesis that the Florencia population consisted of two clusters. We determined the number of ancestral clusters, *K*, using an ad hoc statistic ΔK based on the rate of change in the log probability of data for *K *between 1 and 5 in multiple runs [45]. Each run consisted of 10^6 ^iterations, after a burning period of 10^4 ^iterations. To use these programs, Hardy-Weinberg and linkage equilibrium are assumed, and the software differentiates mixed populations on the basis of allele frequencies at each locus. Finally, population differentiation (F_ST_) and genetic distances (D_A_) among the two Florencia types were calculated with Arlequin v2000 [42].

## Results

**Figure 2:**
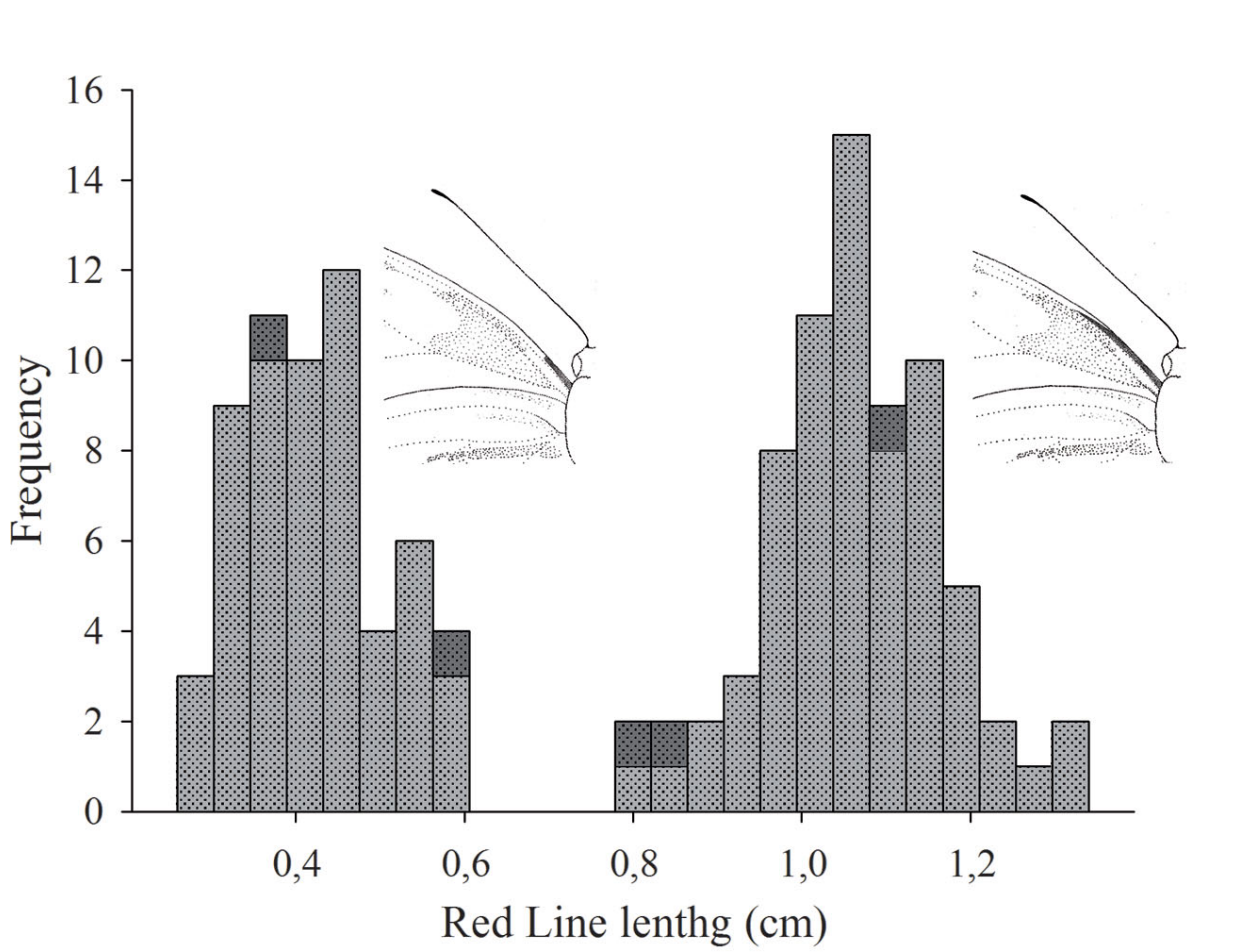
**Histogram of phenotypic measurements of the ventral red line**. The measurements taken from 131 forewings clearly show a bimodal distribution. The short line individuals represent the *H. cydno *cognate and the long line individuals *H. m. malleti*, respectively. In dark gray are the five individuals with intermediate population assignment probabilities determined from microsatellite data.

One of us (ML) originally noticed that specimens collected in Florencia represented two phenotypes, one of which was larger, darker red in colour and with a broader forewing yellow band. However, none of these phenotypic characters proved to be reliably diagnostic (Figure 1). Only the red line, probably homologous to the previously described "red dot" in *H. c. weymeri *[46,47], on the anterior edge of the ventral forewing was identified as a consistent diagnostic character of two morphotypes in the Florencia population. There was a clearly bimodal distribution in the length of this red line, measured relative to the distance between the base of the Discal Cell and its intersection with the Cubital Vein (Cu2) (Figure 2). This character was therefore used to assign individuals to the two morphotypes for further analysis, with the longer red line being diagnostic of *H. melpomene malleti *and the shorter line of the putative *H. cydno *cognate. There was a significant difference in wing size between the two morphotypes, with *H. melpomene malleti *having a smaller wing size similar to other populations analysed (additional file 2). When offspring were raised from field collected females, the colour of the larval head capsule also differed significantly between the *H. m. malleti *and the *H. cydno *cognate individuals from Florencia (additional file 3).

### Species relationships

**Figure 3:**
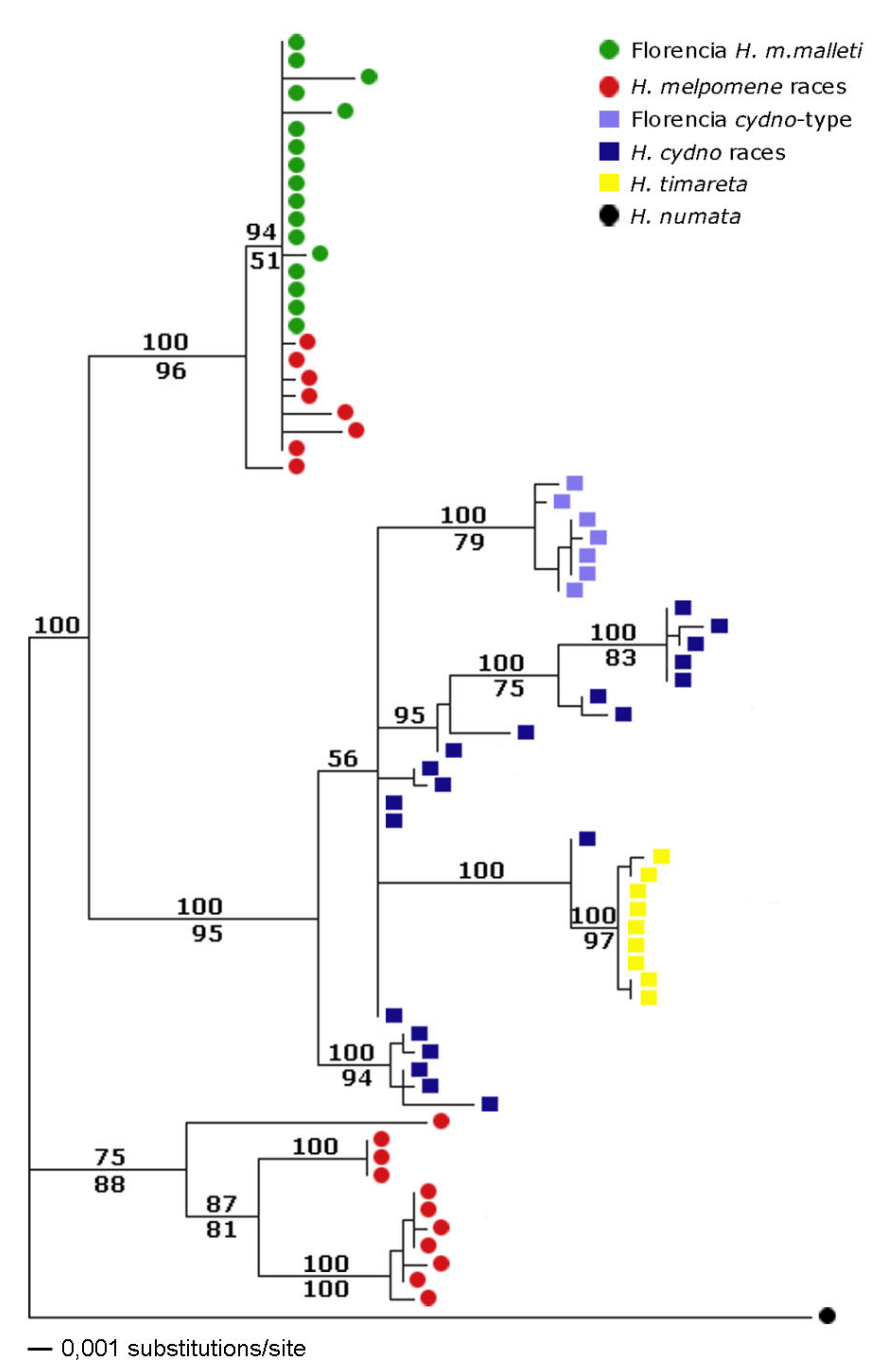
**mtDNA Phylogenetic tree**. Phylogenetic relationships of *H. m. malleti *(green circles) and Florencia *H. cydno *cognate (pale blue squares) with other populations of *H. melpomene *(red circles) and *H. cydno *(dark blue squares) based on CoI and CoII sequences. Also included are specimens of *H. timareta *collected from eastern Ecuador (yellow squares). In additional files (additional file 1) Sequence ID's beginning with AF and AY indicate GenBank accession numbers. Branch lengths and probability values (over branches) were estimated using Bayesian analysis and bootstrap support (under branches) derived from a Maximum Parsimony analysis.

**Figure 4:**
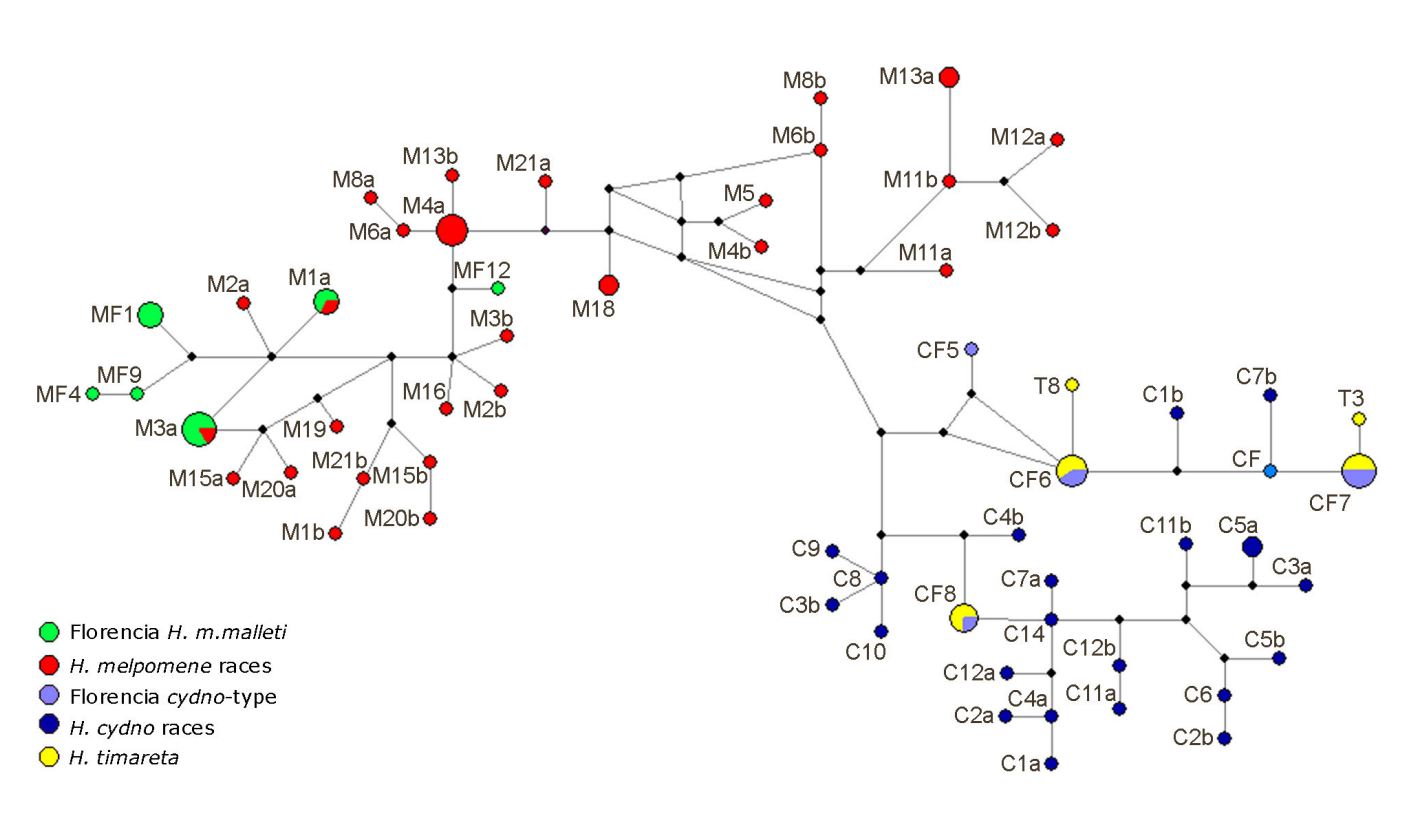
**Allele network for nuclear gene *Tpi***. Red, green, dark blue, light blue and yellow are *H. melpomene *races, *H. m. malleti *from Florencia, *H. cydno *races, *H. cydno *cognate from Florencia and *H. timareta*. Respective alleles are identified with the letters M, MF, C, CF and T, followed by the individual number and allele letter. Black dots are hypothetical ancestors. Sizes of the circles reflect allele frequencies in the population. Networks were constructed with statistical parsimony in TCS v 1.21 [61].

In mtDNA, both parsimony and Bayesian methods produced three well-supported clades (Figure 3): 1) an eastern *melpomene *clade, 2) an *H. cydno *clade including all the *H. cydno *and *H. timareta *sequences and 3) a western *melpomene *clade (Figure 3). Within the *cydno *clade, individuals sampled near to Florencia fell into two different clades corresponding to their phenotypic assignment described above. Individuals with longer red line phenotypes are to the eastern *H. melpomene *and those with the shorter red line form a monophyletic group inside *H. cydno*. The *H. timareta *samples from Ecuador form a distinct monophyletic group also within *H. cydno*. At *Tpi*, two clusters are well resolved with high posterior probability and bootstrap support (99), corresponding to *H. melpomene *and *H. cydno *(1.1% net divergence). The individuals from Florencia fell into the *H. melpomene *and *H. cydno *clades as predicted from their morphological assignment (Figure 4).

### Microsatellite Data Analyses

Individuals from Florencia were screened for variation at thirteen microsatellite loci. The *H. cydno *cognate was less variable, with a mean observed heterozygosity of 0.39 as compared to 0.66 in *H. m. malleti*. All loci were in Hardy-Weinberg equilibrium within the two morphotypes, except *Hm19*, which showed significant heterozygote deficit for both forms, most likely due to null alleles which are known to be present at this locus [48]. All subsequent analyses are presented with this locus removed. The microsatellite F_ST _value between the two morphotypes in Florencia was large and significant (F_ST _= 0.24226, P < 0.0001).

**Figure 5:**
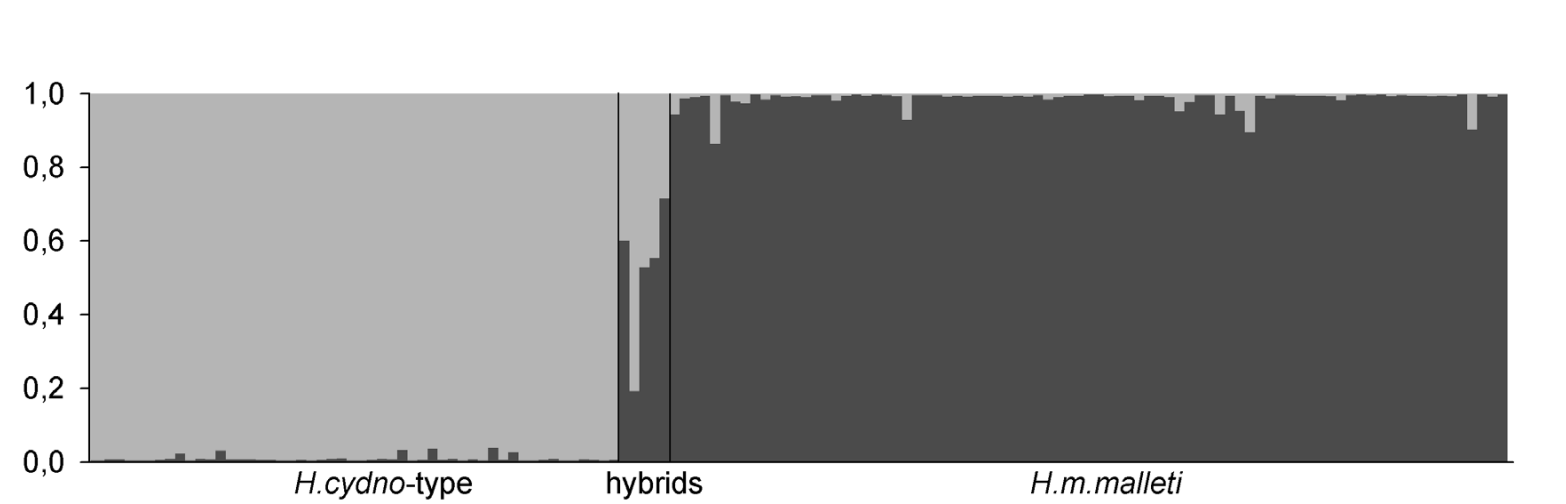
**Assignment test analysis**. Genetic differentiation between *H. m. malleti *and *H. cydno *cognate using assignment analysis of multilocus microsatellite data in Structure 2.2. The relative genome contribution of the two clusters to each individual are shown in dark and light grey.

Without reference to the morphological assignment, two distinct algorithms, STRUCTURE 2.2 and BAPS 4, were used to test the number of distinct populations (K) sampled. The ad-hoc statistic ΔK, estimated using both programs, indicated that the most likely value of K was two (additional file 4). This conclusion was robust to different assumptions made by STRUCTURE (presence or absence of genetic admixture, and correlated or uncorrelated allele frequencies). These two distinct populations identified by STRUCTURE represented the two colour pattern types already identified *a priori*, with virtually all individuals correctly assigned to the appropriate cluster with posterior probabilities = 0.95 (Figure 5).

Five individuals had clearly intermediate assignment probabilities, suggesting possible hybrid genotypes. In support of this hypothesis, these individuals also had some evidence for colour pattern introgression, with either somewhat intermediate red line phenotypes (Figure 2) or melanic scales in the forewing band. These individuals represented around 3.5% of the total sample (5/142) with three of the five being consistent with an F_1 _genotype (Figure 5). Five additional individuals in the *H. m. malleti *cluster showed posterior probabilities less than 0.95 suggesting that these might represent backcross hybrids.

### No-choice mate experiments

A total of 112 trials were performed (Table 1). An initial null model with a single mating probability (a = b = c = d) was established across all trials (LnL = -88.5821). To test different hypotheses, the likelihood model was fitted in a stepwise manner by adding parameters to the initial model. When mating probabilities were estimated separately for intra and inter morphotypes (2p: a = d and b = c) this led to a significant improvement in fit of the model (G = 119.73, d.f. = 1 and p < 0.0001). This reflects the different mating frequencies between inter and intra morphotype trials (3.5% and 96.5%, respectively; Table 1). A more complicated four parameter model with asymmetric mating probabilities did not significantly improve fit of the model (4p: a≠b≠c≠d, G = 4.74, d.f. = 2 and p > 0.09).

**Table 1:** Results from the no-choice mating trials

female		male	m	n	t	Mating probability	C.I (Upper)	C.I. (Lower)	Parameters
*H.m. malleti*	x	*H.m. malleti*	21	1	22	0.9545	0.9594	0.9506	*a*
***H.m. malleti***	**×**	***H. cydno-*type**	**1**	**26**	**27**	**0.0370**	**0.0420**	**0.0246**	***b***
***H. cydno-*type**	**×**	***H.m. malleti***	**1**	**23**	**24**	**0.0417**	**0.0714**	**0.0000**	***c***
*H. cydno-*type	×	*H. cydno-*type	35	4	39	0.8974	0.8869	0.9313	*d*

Mating probabilities were estimated using likelihood as described in the text, with upper and lower confidence intervals shown. *m*: number of trials with mating occurring; *n*: number of trials without mating occurring; *t*: total number of trials.

### Host plant preference

Nineteen females from Florencia (10 *H. m. malleti *and 9 *H. cydno*-type) were assessed for host preference. Females assigned as *H. m. malleti *used primarily two plants (471 eggs in total on *P. oerstedii*, 71%, and *P. ligularis*, 28%), while the *H. cydno *cognate females oviposited on many species (729 eggs in total on *P. edulis *48%, *P. ligularis *15%, *P. oerstedii *22%, and *P. quadrangularis*, *P. arborea *and *P. maliformis *14%).

## Discussion

The colour patterns of *H. melpomene *and *H. cydno *are traits under strong ecological selection that also contribute to speciation [12,23,26,49,50]. Divergence in mimetic pattern contributes to reproductive isolation by assortative mating, due to the use of colour as a mate recognition signal and by frequency dependent mimicry selection against rare colour pattern hybrids [12,23,26,29]. The fact that such a major role for mimicry in the reproductive isolation of currently hybridizing species has been clearly demonstrated has led to an expectation that mimicry is unlikely between closely related species with incomplete reproductive isolation [15]. The results of the present study clearly show that this is not always the case. The butterflies collected in Florencia represent two mimetic species in the *H. melpomene *and *H. cydno *clades respectively. Despite the extreme phenotypic similarity between the butterflies studied here, the data indicate clear concordance between nuclear, mtDNA and phenotypic markers in assigning over 90% of the individuals sampled to one or the other morphotype. These represent a population of the widespread race *H. m. malleti*, and a novel entity related to *H. cydno*. Nonetheless, there is clear evidence for ongoing interspecific hybridization with around 3.5% of individuals sampled representing clearly identifiable hybrids.

The strong concordance between markers and relative scarcity of hybrids implies strong reproductive isolation. The mating experiments described here demonstrate strong assortative mating, and host choice experiments further imply some degree of ecological isolation. The strength of assortative mating in our experiments is actually greater than that between the phenotypically very divergent *H. melpomene melpomene *and *H. cydno cordula *(≈ 82%, Mavárez et al. 2006). It therefore seems likely that there has been increased divergence in mating signals apart from colour – most likely pheromonal – to allow the Florencia species to coexist. As colour is used as a cue in mate finding, we would predict that males are likely to be attracted to the pattern of the "wrong" species, but that this is compensated for by divergence in other mating signals.

*H. melpomene *and *H. cydno *are known to differ in habitat preference and host use [30,51]. Our host choice data imply that this ecological difference is maintained between the Florencia species, with the *H. cydno *cognate more of a host generalist similar to other populations of *H. cydno*. It seems likely that this corresponds to a preference for forest habitats, as is the case for other *H. cydno *populations, leading to ecological isolation. Although crosses between the Florencia forms have not been carried out, there is preliminary evidence that the *H. cydno *cognate morphotype is compatible with other populations of *H. cydno*, while the Florencia *H. m. malleti *shows hybrid sterility with *H. cydno *(Giraldo and Linares, unpub). It seems likely that the Florencia species show similar female hybrid sterility as compared to other sympatric *H. melpomene *and *H. cydno *populations.

In order to clarify the role of colour pattern in speciation we need to consider the order of divergence in different factors. Divergence might have occurred initially in factors other than colour pattern, such as habitat preference and pheromonal mating cues. Divergence in mimicry then occurred subsequent to speciation in most populations of the *H. cydno *group, except those in the eastern Andes such as that studied here. Alternatively, speciation might have been initially triggered by divergence in colour pattern with mimetic convergence a derived state acquired subsequent to speciation in the Florencia region. The first explanation is more parsimonious with respect to colour pattern, but may actually be less likely. The derived position of the *H. cydno *cognate form in the mtDNA phylogeny suggests that it has evolved from a more *H. cydno *like ancestor (Figure 3). Furthermore, the observation that intraspecific races of *H. melpomene *show both divergent colour patterns and strong assortative mating implies that mimicry and associated mate preferences are the first steps in divergence in this group [13].

Under the second scenario, an initial divergence in colour pattern associated with adaptation to different Müllerian mimicry rings became associated with further changes in ecology and hybrid inviability, eventually leading to speciation [12]. Subsequently, strong mimetic selection in the sympatric Florencia population has led to convergence of *H. cydno *onto the *H. melpomene *pattern. This might have occurred through adaptive introgression of colour pattern genes between the species [21,23,26,52-54]. It seems plausible that the lack of suitable mimicry models in the *H. sapho *group has led this east Andean population to secondarily converge on the *H. melpomene *pattern.

**Figure 6:**
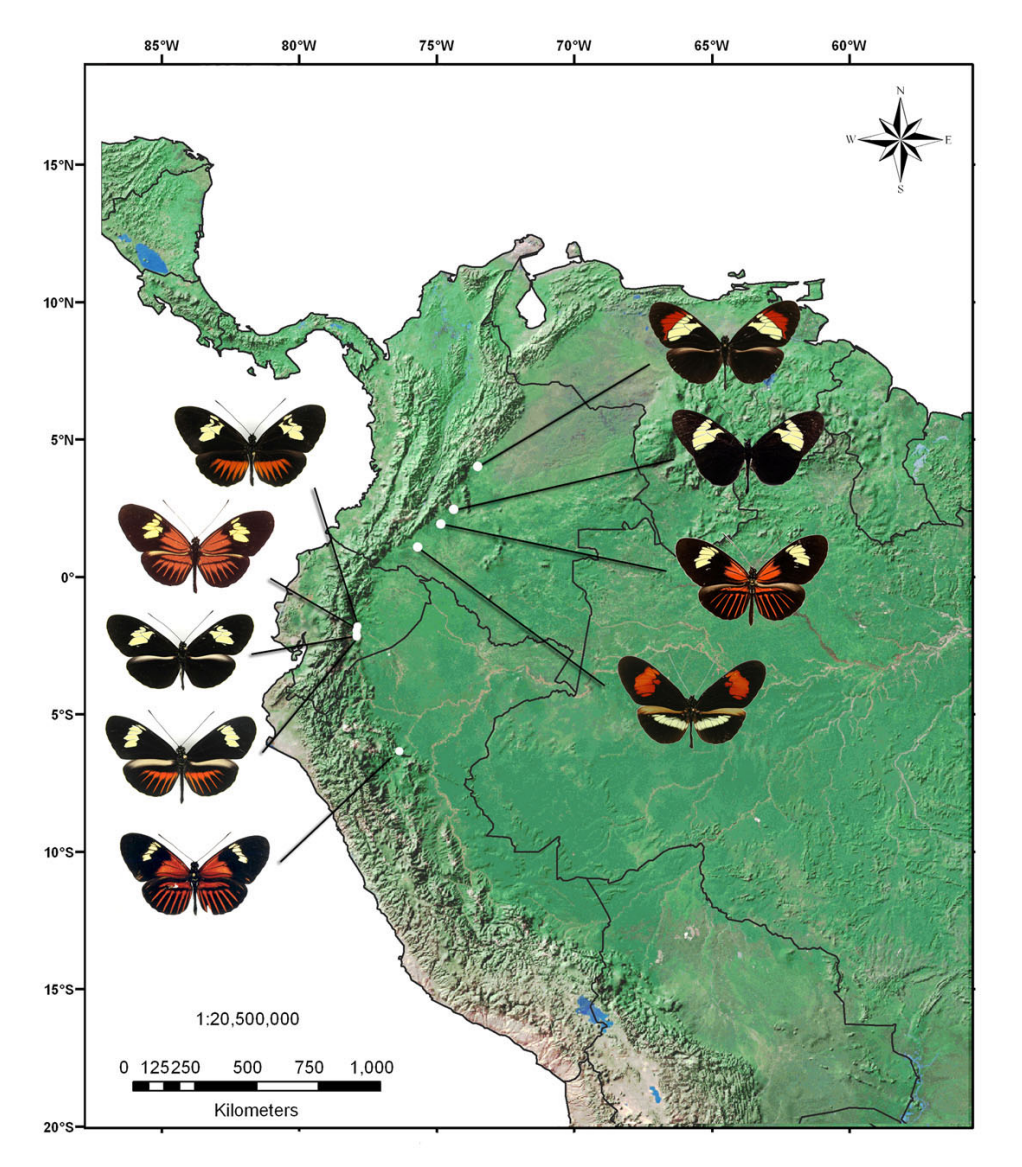
**East Andean geographical distribution of known *H. cydno *clade taxa**. On the left from top to bottom: *H. t. timareta f. contigua*, *H. t. timareta f. timareta*, *H. t. timareta f. timareta*, *H. t. timareta f. peregrina *and *H. t. timoratus *(Lamas 1998). On the right: *H. heurippa*, *H. cydno *cognate from Rio Pato (undescribed), *H. t. florencia*, *H. tristero*. See text for details.

Our results raise the possibility that other populations of cryptic species might exist in *Heliconius*. Notably the cryptic *H. cydno *cognate, *H. tristero *which was described based on two specimens and shares a red and yellow banded colour pattern with sympatric *H. m. mocoa *in Putumayo, Colombia may represent another example of the same phenomenon [25] (Figure 6). Another potential case occurs in Peru, where a "postman" *H. cydno *has been discovered that mimics *H. m. amaryllis *(Mavaréz et al. unpub.).

The new *H. cydno *cognate from Florencia resembles *H. timareta*, a known relative of *H. cydno *from eastern Ecuador. *H. timareta *is polymorphic with one orange rayed form that is very similar to the taxon from Florencia (Figure 6). Furthermore, a recently described subspecies, *H. t. timoratus *Lamas (1998) from Perú is a near perfect mimic of *H. m. malleti*, although the two have so far not been collected in sympatry (Figure 6). Due to the colour pattern similarity with *H. t. timoratus *of Perú and the east Andean distribution of both forms, here we propose the *H. cydno *cognate from Florencia as a new northern race of *H. timareta*, which forms a mimicry ring with *H. m. malleti *and *H. e. lativitta *in the southern foothills of Colombia. We propose to name this form after Florencia, the town where it was discovered. From a geographical point of view (Figure 6) the *H. cydno *clade in the eastern Andes is represented by, from north to south, *H. cydno cordula*, *H. heurippa*, *H. timareta florencia *and *H. tristero *in Colombia; *H. timareta timareta *in Ecuador and *H. timareta timoratus *and another new *H. cydno*-like taxon in Perú (Mavarez et al., in prep.).

*H. timareta florencia *is anatomically similar to *H. timareta *and *H. cydno *races, with a FW area of 5,26 to 6,64 cm^2 ^(additional file 2). The FW has two principal pattern elements in a black background, 1. An irregular yellow postmedial band extending proximodistally from distal end of discal cell to R_2_–R_3 _fork, and laterally from subcostal to CU_1a_, and 2. A red "Dennis" element extending from the basal end of the discal cell to the CU_1b_-discal cell fork, which is generally shorter than the *H. m. malleti *FW element (the approximate basal fourth of the FW). The ventral FW is similar with a slight reduction in the yellow band area and the distinguishing red line with length 2,65 to 5,92 mm (Figure 1b and Figure 2). The HW also has a black background colour and an androconial distribution as in *H. timareta*. A reddish dennis-ray element [55] is similar in form to the dennis-ray in *H. m. malleti*, with a narrow red longitudinal bar D (Dennis) and six red radiate marks on the discal part of the upper side [55]. The ventral HW underside is similar with narrower red areas. Males and females are phenotypically similar. *H. t. florencia *is registered in ZooBank with the unique digital identifier LSID: 49656F41-E817-4FD6-B28D-6AA362CC5268. In order to comply with the International Code of Zoological Nomenclature, paper copies of this electronic article have been deposited in the following libraries: The Entomology Library of the Natural History Museum, London, UK; The Balfour Library, Department of Zoology, University of Cambridge, UK; The Genetics Library, Department of Genetics, University of Cambridge, UK; Biblioteca Universidad Nacional de Colombia, Hemeroteca Nacional, Sede Bogotá; Biblioteca Universidad de los Andes, Bogotá, Colombia.

*Type material*: The holotype male specimen is deposited in the permanent collection of the Natural History Museum ANDES of Universidad de los Andes in Colombia. The data label reads: Andes-E-11517, Colombia, Caquetá, Florencia, Quebrada las Doraditas, 01/09/2008. The red holotype label reads: Holotipo, Andes-E-11517, *Heliconius timareta florencia*.

## Conclusion

Obviously the study of cryptic species has a long history and, especially with the advent of modern molecular techniques many previously undescribed taxa have been detected [56]. However, cryptic species often use sensory modalities that humans do not readily perceive, such as pheromones [57,58], toxicity resistance [59] and imperceptible song differences [60]. The surprising aspect of this study is therefore the discovery of cryptic species in well-studied taxa where speciation is commonly triggered by bright visual signals. It is increasingly becoming clear that tropical biodiversity is severely underestimated and that combining morphological and DNA sequence analysis is a powerful tool for species discovery. Finally, our mate and host choice experiments suggest that the sympatric species in Florencia combine the same ecological differences known to occur in other parts of the range of *H. cydno *and *H. melpomene *with enhanced pheromonal signals to compensate for the lack of colour pattern signals. Our results therefore highlight the fact that speciation is the combined result of divergence along multiple phenotypic axes.

## Authors' contributions

NG carried out laboratory work, mating experiments, plant choice trials, larvae and adult morphology measures and description, and behavioural, morphological and genetic analyses. NG and CDJ designed experiments. CS contributed with mtDNA sequences of *Heliconius timareta *from Ecuador and genetic analysis. CDJ helped in data analysis and, with NG and CS, drafted the manuscript. EB and ML participated in the design and coordination. ML conceived the study, did the field observations and obtained all the specimens from Florencia used in this study. All authors read and approved the final manuscript.

## Supplementary Material

Additional file 1 **Individuals used in phylogenetic analyses**. Gene accession number and locality of alleles and individuals included in the phylogenetic analysis. Click here for file

Additional file 2 **Forewing size between *H. cydno *and *H. melpomene***. Wild individuals from Florencia and museum specimens of *H. cydno *and *H. melpomene *were measured for forewing area. Wings were scanned next to a ruler and processed in *tpsUtil *and *tpsDig *9 image software to measure area. Tests for normal distribution were carried out for each species and a one-way Analysis of Variance (ANOVA) by species and sex was performed in SPSS to test for differences in size. Tukey's post-hoc was used to compare median values between pairwise population sets. *H. m. malleti *did not differ in wing area from other *H. melpomene *races (ANOVA p > 0,05). *H. cydno *cognate wing area is similar to *H. cydno *races (ANOVA p > 0,05). Click here for file

Additional file 3 **Larvae colour dispersion index b' based on intensity and brightness of the head capsule**. *H. melpomene *has a light dust cephalic tone with dark and broad bands, while *H. cydno *has a dark yellow-orange cephalic tone and light narrow bands. b' = b/(r+g+b) based on RGB filters using the procedure as in Endler et al. 1990. *H. cydno *races are: *H. c. cordula*, *H. c. cydnides *and *H. c. zelinde *and *H. melpomene *races are *H. m. mocoa *and *H. m. vulcanus*. Analysis of variance shows high differences between *melpomene *and *cydno *groups (*H. melpomene *and *H. m. malleti *vs. *H. cydno *and *H. cydno *cognate; p < 0,0001). Click here for file

Additional file 4 **Best cluster assignment**. Magnitude of ΔK as a function of K (mean ± SD over 5 replicates), calculated using the procedure of Evanno et al. (2005). *a*) ΔK for Structure (Ln for K = 2:-4691,42) and *b*) ΔK for BAPS 4 (Ln for K = 2:-4987,95). Click here for file
