# Supplementary material for: Two sisters in the same dress: Heliconius cryptic species
Source: BMC Evol Biol. 2008 Nov 28;8:324. doi: 10.1186/1471-2148-8-324 (PMC2632674; doi:10.1186/1471-2148-8-324)
Supplement: Additional file 1 — Individuals used in phylogenetic analyses. Gene accession number and locality of alleles and individuals included in the phylogenetic analysis. [file 1471-2148-8-324-S1.doc]

| **Species** | **subspecies** | **individual** | **Tpi allele** | **name in Tpi net** | **Genebank Tpi** | **Genebank CO** | **locality** |
| --- | --- | --- | --- | --- | --- | --- | --- |
| *H.melpomene* | *mocoa* | M155 | 1 | M1a | DQ019241 | AY548136 | Mocoa, Colombia |
| *H.melpomene* | *mocoa* | M155 | 2 | M1b | DQ019242 |  | Mocoa, Colombia |
| *H.melpomene* | *mocoa* | M67 | 1 | M2a | AY548146 | AY548120 | Mocoa, Colombia |
| *H.melpomene* | *mocoa* | M67 | 2 | M2b | AY548149 |  | Mocoa, Colombia |
| *H.melpomene* | *mocoa* | M74 | 1 | M3a | AY548147 | AY548125 | Mocoa, Colombia |
| *H.melpomene* | *mocoa* | M74 | 2 | M3b | AY548141 |  | Mocoa, Colombia |
| *H.melpomene* | *mocoa* | M78 |  |  |  | AY548128 | Mocoa, Colombia |
| *H.melpomene* | *rosina* | M544 | 1 | M4a | AF413789 | AF413674 | Panamá |
| *H.melpomene* | *rosina* | M544 | 2 | M4b | AF413790 |  | Panamá |
| *H.melpomene* | *rosina* | M533 | 1 | M5 | AF545457 | AF512987 | Panamá |
| *H.melpomene* | *rosina* | M532 | 1 | M6a | AF545455 | AF512988 | Panamá |
| *H.melpomene* | *rosina* | M532 | 2 | M6b | AF545456 |  | Panamá |
| *H.melpomene* | *rosina* | M546 | 1 | M7 | AF545452 | AF512982 | Panamá |
| *H.melpomene* | *rosina* | M811 | 1 | M8a | AF413781 | AF413673 | Panamá |
| *H.melpomene* | *rosina* | M811 | 2 | M8b | AF413782 |  | Panamá |
| *H.melpomene* | *cythera* | 8073 | 1 | M9a | AF413779 | AF413678 | Ecuador |
| *H.melpomene* | *cythera* | 8073 | 2 | M9b | AF413780 |  | Ecuador |
| *H.melpomene* | *cythera* | 8074 | 1 | M10 | AF413781 | AF413677 | Ecuador |
| *H.melpomene* | *melpomene* | M415 | 1 | M11a | AF545465 | AF512975 | French Guyana |
| *H.melpomene* | *melpomene* | M415 | 2 | M11b | AF545466 |  | French Guyana |
| *H.melpomene* | *melpomene* | M436 | 1 | M12a | AF413774 | AF413675 | French Guyana |
| *H.melpomene* | *melpomene* | M436 | 2 | M12b | AF413775 |  | French Guyana |
| *H.melpomene* | *melpomene* | M437 | 1 | M13a | AF413791 | AF413676 | French Guyana |
| *H.melpomene* | *melpomene* | M437 | 2 | M13b | AF413792 |  | French Guyana |
| *H.melpomene* | *melpomene* | M507 | 1 | M14 | AF545460 | AF512986 | French Guyana |
| *H.melpomene* | *amaryllis* | JM1917 | 1 | M15a | AY329833 |  | Perú |
| *H.melpomene* | *amaryllis* | JM1917 | 2 | M15b | AY329834 |  | Perú |
| *H.melpomene* | *amaryllis* | JM1271 | 1 | M16 | AY329832 |  | Perú |
| *H.melpomene* | *amaryllis* | 04-288 |  |  |  | AM709831 | Perú |
| *H.melpomene* | *malleti* | 8158 | 1 | M17 | AY548152 | AY548140 | Perú |
| *H.melpomene* | *malleti* | 11444 | 1 | M18 | EU852063 | | Perú |
| *H.melpomene* | *aglaope* | JM1178 | 1 | M19 | AY329831 |  | Perú |
| *H.melpomene* | *aglaope* | JM1174 | 1 | M20a | AY329829 |  | Perú |
| *H.melpomene* | *aglaope* | JM1174 | 2 | M20b | AY329830 |  | Perú |
| *H.melpomene* | *aglaope* | JM494 | 1 | M21a | AY329827 |  | Perú |
| *H.melpomene* | *aglaope* | JM494 | 2 | M21b | AY329828 |  | Perú |
| *H.melpomene* | *aglaope* | 04-286 |  |  |  | AM709829 | Perú |
| *H.melpomene* | *aglaope* | 04-288 |  |  |  | AM709830 | Perú |
| *H.cydno* | *chioneus* | STRI-B-569 | 1 | C1a | AF545441 | AF512989 | Panamá |
| *H.cydno* | *chioneus* | STRI-B-569 | 2 | C1b | AF545442 |  | Panamá |
| *H.cydno* | *chioneus* | STRI-B-559 | 1 | C2a | AF545439 | AF512991 | Panamá |
| *H.cydno* | *chioneus* | STRI-B-559 | 2 | C2b | AF545440 |  | Panamá |
| *H.cydno* | *chioneus* | STRI-B-566 | 1 | C3a | AF545445 | AF512993 | Panamá |
| *H.cydno* | *chioneus* | STRI-B-566 | 2 | C3b | AF545446 |  | Panamá |
| *H.cydno* | *chioneus* | STRI-B-567 | 1 | C4a | AF545443 | AF512990 | Panamá |
| *H.cydno* | *chioneus* | STRI-B-567 | 2 | C4b | AF545444 |  | Panamá |
| *H.cydno* | *chioneus* | STRI-B-552 | 1 | C5a | AF545449 | AF512985 | Panamá |
| *H.cydno* | *chioneus* | STR-B-552 | 2 | C5b | AF545450 |  | Panamá |
| *H.cydno* | *chioneus* | STRI-B-560 | 1 | C6 | AF545447 | AF512992 | Panamá |
| *H.cydno* | *weymeri* | M19 | 1 | C7a | AY548142 | AY548115 | Valle del Cauca, Colombia |
| *H.cydno* | *weymeri* | M19 | 2 | C7b | AY548143 |  | Valle del Cauca, Colombia |
| *H.cydno* | *weymeri* | M18 |  |  |  | AY548114 | Valle del Cauca, Colombia |
| *H.cydno* | *weymeri* | M20 |  |  |  | AY548116 | Valle del Cauca, Colombia |
| *H.cydno* | *galanthus* | TX511 | 1 | C8 | DQ448499 | AY744622 | Costa Rica |
| *H.cydno* | *galanthus* | TX514 | 1 | C9 | DQ448500 | AY744625 | Costa Rica |
| *H.cydno* | *galanthus* | TX515 | 1 | C10 | DQ448501 | AY744626 | Costa Rica |
| *H.cydno* | *galanthus* | TX630 |  |  |  | AY744604 | Costa Rica |
| *H.cydno* | *galanthus* | TX631 |  |  |  | AY744605 | Costa Rica |
| *H.cydno* | *galanthus* | TX624 |  |  |  | AY744606 | Costa Rica |
| *H.cydno* | *cordula* | M101 | 1 | C11a | DQ019234 | DQ019244 | San Cristobal, Venezuela |
| *H.cydno* | *cordula* | M101 | 2 | C11b | DQ019235 |  | San Cristobal, Venezuela |
| *H.cydno* | *cordula* | M104 | 1 | C12a | DQ019236 | DQ019245 | San Cristobal, Venezuela |
| *H.cydno* | *cordula* | M104 | 2 | C12b | DQ019237 |  | San Cristobal, Venezuela |
| *H.cydno* | *cordula* | M187 | 1 | C13 | DQ019238 | DQ019251 | San Cristobal, Venezuela |
| *H.cydno* | *cordula* | M182 | 1 | C14 | DQ019239 | DQ019250 | San Cristobal, Venezuela |
| *H.cydno* | *cordula* | M105 |  |  |  | DQ019246 | San Cristobal, Venezuela |
| *H.timareta* |  | STRI-B-8520 | | T1 | EU852085 | AY748060 | Ecuador |
| *H.timareta* |  | STRI-B-8521 | | T2a | EU852086 | AY748028 | Ecuador |
| *H.timareta* |  | STRI-B-8521 | | T2b | EU852087 | | Ecuador |
| *H.timareta* |  | STRI-B-11436 | | T3 | EU852088 | EU848524 | Ecuador |
| *H.timareta* |  | STRI-B-11413 | | T4 | EU852089 | EU848525 | Ecuador |
| *H.timareta* |  | STRI-B-8529 | | T5 | EU852090 | EU848526 | Ecuador |
| *H.timareta* |  | STRI-B-11432 | | T6 | EU852091 | EU848527 | Ecuador |
| *H.timareta* |  | STRI-B-11439 | | T7 | EU852092 | EU848528 | Ecuador |
| *H.timareta* |  | STRI-B-8533 | | T8a | EU852093 | EU848529 | Ecuador |
| *H.timareta* |  | STRI-B-8533 | | T8b | EU852094 | | Ecuador |
| *H.timareta* |  | STRI-B-8523 | | T9 | EU852095 | EU848530 | Ecuador |
| *H.numata* |  | STRI-B-346 |  |  | AF413773 | AF413681 | French Guyana |
| *H.melpomene* | *malleti* | M272 |  | MF1 | EU852064 | EU848500 | Sucre, Florencia, Colombia |
| *H.melpomene* | *malleti* | M430 |  | MF2 | EU852065 | EU848501 | Sucre, Florencia, Colombia |
| *H.melpomene* | *malleti* | M437 |  | MF3 | EU852066 | EU848502 | Sucre, Florencia, Colombia |
| *H.melpomene* | *malleti* | M438 |  | MF4 | EU852067 | EU848503 | Sucre, Florencia, Colombia |
| *H.melpomene* | *malleti* | M454 |  | MF5 | EU852068 | EU848504 | Sucre, Florencia, Colombia |
| *H.melpomene* | *malleti* | M459 |  | MF6 | EU852069 | EU848505 | Sucre, Florencia, Colombia |
| *H.melpomene* | *malleti* | M470 |  |  |  | EU848506 | Sucre, Florencia, Colombia |
| *H.melpomene* | *malleti* | M502 |  | MF7 | EU852070 | EU848507 | Sucre, Florencia, Colombia |
| *H.melpomene* | *malleti* | M503 |  |  |  | EU848508 | Sucre, Florencia, Colombia |
| *H.melpomene* | *malleti* | M510 |  | MF8 | EU852071 | EU848509 | Sucre, Florencia, Colombia |
| *H.melpomene* | *malleti* | M512 |  | MF9 | EU852072 | EU848510 | Sucre, Florencia, Colombia |
| *H.melpomene* | *malleti* | M579 |  | MF10 | EU852073 | EU848511 | Sucre, Florencia, Colombia |
| *H.melpomene* | *malleti* | M594 |  |  |  | EU848512 | Sucre, Florencia, Colombia |
| *H.melpomene* | *malleti* | M702 |  | MF11 | EU852074 | EU848513 | Sucre, Florencia, Colombia |
| *H.melpomene* | *malleti* | M704 |  | MF12 | EU852075 | EU848514 | Sucre, Florencia, Colombia |
| *H.melpomene* | *malleti* | M707 |  | MF13 | EU852076 | EU848515 | Sucre, Florencia, Colombia |
| *H.melpomene* | *malleti* | M1002 |  |  |  | EU848516 | Sucre, Florencia, Colombia |
| *H.timareta* | *florencia* | M326 |  | CF1 | EU852077 | | Sucre, Florencia, Colombia |
| *H.timareta* | *florencia* | M399 |  | CF2 | EU852078 | EU848517 | Sucre, Florencia, Colombia |
| *H.timareta* | *florencia* | M419 |  | CF3 | EU852079 | EU848518 | Sucre, Florencia, Colombia |
| *H.timareta* | *florencia* | M436 |  | CF4 | EU852080 | EU848519 | Sucre, Florencia, Colombia |
| *H.timareta* | *florencia* | M473 |  | CF5 | EU852081 | EU848520 | Sucre, Florencia, Colombia |
| *H.timareta* | *florencia* | M486 |  |  |  | EU848521 | Sucre, Florencia, Colombia |
| *H.timareta* | *florencia* | M504 |  | CF6 | EU852082 | | Sucre, Florencia, Colombia |
| *H.timareta* | *florencia* | M684 |  | CF7 | EU852083 | | Sucre, Florencia, Colombia |
| *H.timareta* | *florencia* | M703 |  |  |  | EU848522 | Sucre, Florencia, Colombia |
| *H.timareta* | *florencia* | M705 |  | CF8 | EU852084 | | Sucre, Florencia, Colombia |
| *H.timareta* | *florencia* | M1009 |  |  |  | EU848523 | Sucre, Florencia, Colombia |
|  |  |  |  |  |  |  |  |
