# Supplementary figures and images for: Two sisters in the same dress: Heliconius cryptic species
Source: BMC Evol Biol. 2008 Nov 28;8:324. doi: 10.1186/1471-2148-8-324 (PMC2632674; doi:10.1186/1471-2148-8-324)

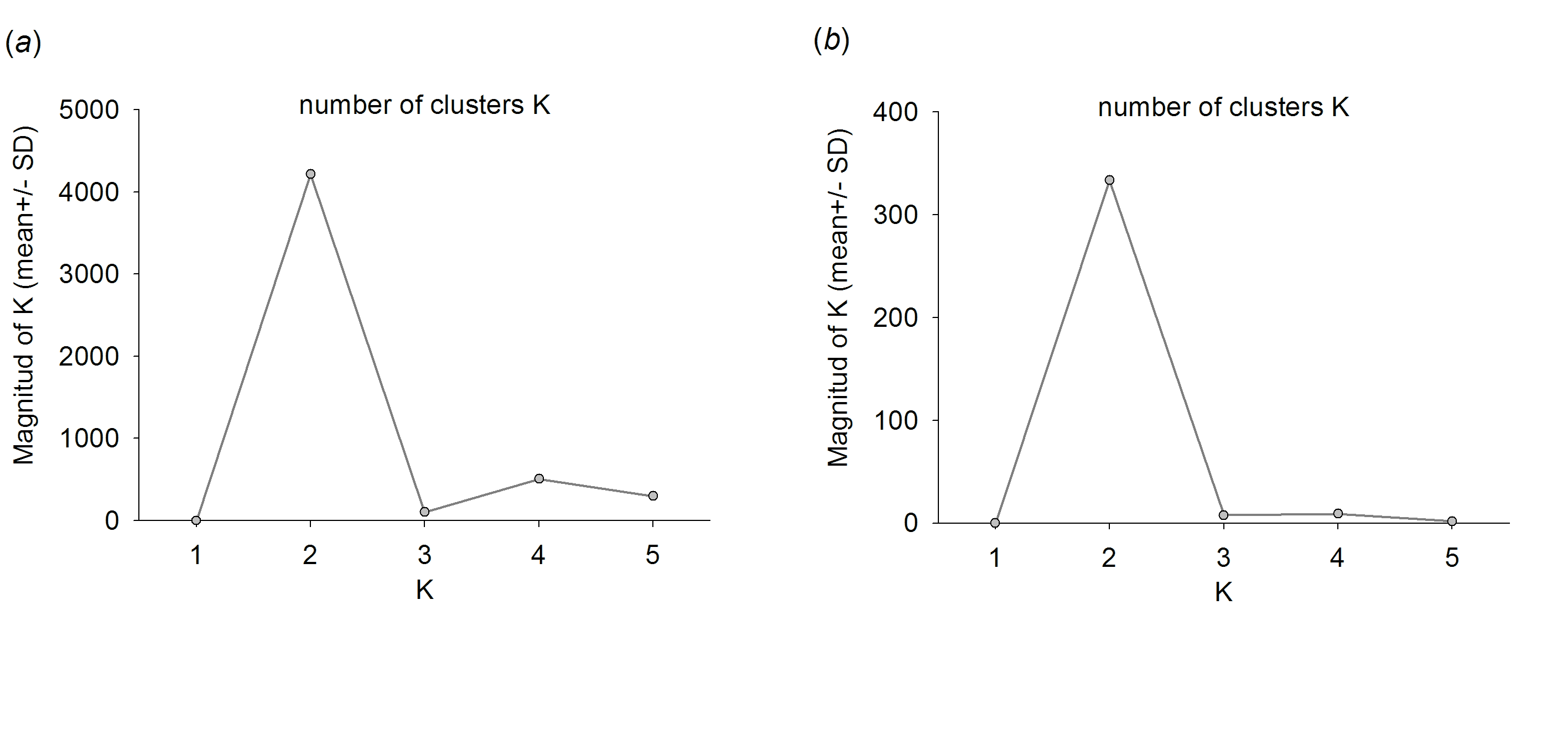

Supplement: Additional file 4 — Best cluster assignment. Magnitude of ΔK as a function of K (mean ± SD over 5 replicates), calculated using the procedure of Evanno et al. (2005). a) ΔK for Structure (Ln for K = 2:-4691,42) and b) ΔK for BAPS 4 (Ln for K = 2:-4987,95). [file 1471-2148-8-324-S4.tiff]
